# The London Dissector, or Guide to Anatomy, for the Use of Students

**Published:** 1842-10-15

**Authors:** 


*The London Dissector, or Guide to Anatomy, for the use of Students. From the last London Edition.* Revised and corrected by Edward J. Chaisty, M. D., late Demonstrator of Anatomy in the University of Maryland. Philadelphia, Barrington & Haswell, 1842. 1 vol. 12mo. pp. 273.

We are glad to see that Messrs. Barrington and Haswell have issued a second edition of this useful little work, which we recommend as an excellent manual to the student of practical anatomy.

---

*Report of the Joint Special Committee, on the Subject of the Effects of Leaden Pipes upon Well-water, in the city of Lowell.* Pages 21.

An elaborate and interesting report, by Dr. Samuel L. Dana.

